# Exploration of phenolic acid derivatives as inhibitors of SARS-CoV-2 main protease and receptor binding domain: potential candidates for anti-SARS-CoV-2 therapy

**DOI:** 10.3389/fchem.2023.1251529

**Published:** 2023-09-26

**Authors:** Nusrat Shafiq, Aiman Mehroze, Warda Sarwar, Uzma Arshad, Shagufta Parveen, Maryam Rashid, Ariba Farooq, Naila Rafiq, Gezahign Fentahun Wondmie, Yousef A. Bin Jardan, Simone Brogi, Mohammed Bourhia

**Affiliations:** ^1^ Synthetic and Natural Products Discovery (SNPD) Laboratory, Department of Chemistry, Government College Women University Faisalabad, Faisalabad, Pakistan; ^2^ Department of Chemistry, University of Lahore, Lahore, Pakistan; ^3^ Department of Biochemistry, Government College Women University Faisalabad, Faisalabad, Pakistan; ^4^ Department of Biology, Bahir Dar University, Bahir Dar, Ethiopia; ^5^ Department of Pharmaceutics, College of Pharmacy, King Saud University, Riyadh, Saudi Arabia; ^6^ Department of Pharmacy, Pisa University, Pisa, Italy; ^7^ Department of Chemistry and Biochemistry, Faculty of Medicine and Pharmacy, Ibn Zohr University, Laayoune, Morocco

**Keywords:** COVID-19, SARS-CoV-2, phenolic acids, molecular docking, density functional theory

## Abstract

Severe acute respiratory Syndrome-Coronavirus-2 (SARS-CoV-2) is the etiological virus of Coronavirus Disease 2019 (COVID-19) which has been a public health concern due to its high morbidity and high mortality. Hence, the search for drugs that incapacitate the virus via inhibition of vital proteins in its life cycle is ongoing due to the paucity of drugs in clinical use against the virus. Consequently, this study was aimed at evaluating the potentials of natural phenolics against the Main protease (Mpro) and the receptor binding domain (RBD) using molecular modeling techniques including molecular docking, molecular dynamics (MD) simulation, and density functional theory (DFT) calculations. To this end, thirty-five naturally occurring phenolics were identified and subjected to molecular docking simulation against the proteins. The results showed the compounds including rosmarinic acid, cynarine, and chlorogenic acid among many others possessed high binding affinities for both proteins as evident from their docking scores, with some possessing lower docking scores compared to the standard compound (Remdesivir). Further subjection of the hit compounds to drug-likeness, pharmacokinetics, and toxicity profiling revealed chlorogenic acid, rosmarinic acid, and chicoric acid as the compounds with desirable profiles and toxicity properties, while the study of their electronic properties via density functional theory calculations revealed rosmarinic acid as the most reactive and least stable among the sets of lead compounds that were identified in the study. Molecular dynamics simulation of the complexes formed after docking revealed the stability of the complexes. Ultimately, further experimental procedures are needed to validate the findings of this study.

## Graphical Abstract

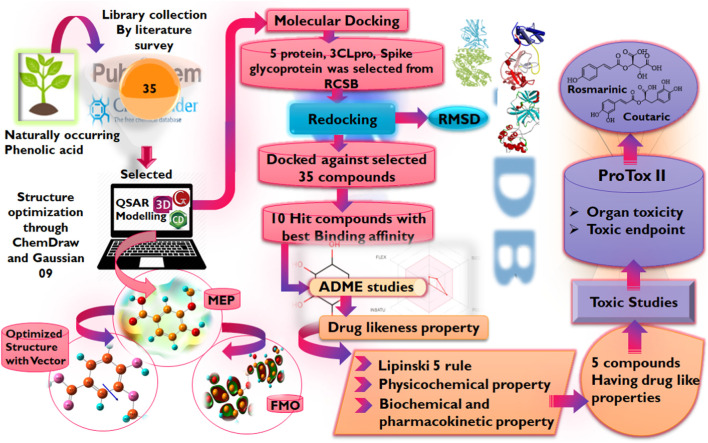

## 1 Introduction

**FIGURE 1:**
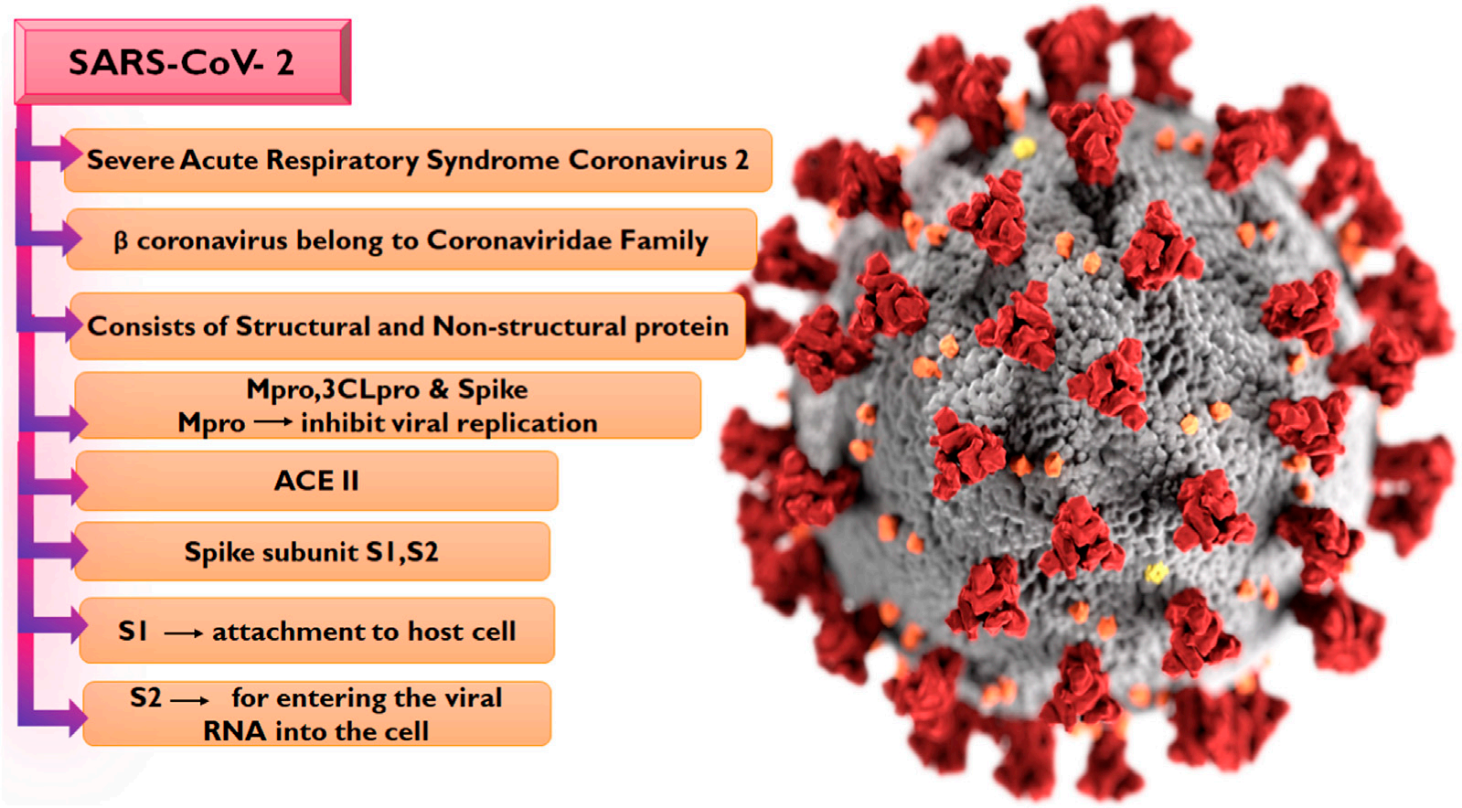
Taxonomy of SARS-CoV-2 and RNA sequencing.

The emergence of the SARS-CoV-2 which is the causative organism of COVID-19 in December 2019 in Wuhan, China was an unprecedented event that would later result in a global outbreak. Interestingly, SARS-CoV-2 exhibits strong genetic ties to bat coronavirus, phylogenetic studies show similarities with the bat coronavirus isolate RaTG13 (Poterico and Mestanza, 2020), and they both belong to the beta coronavirus family along with the Middle East respiratory syndrome (MERS) and severe acute respiratory syndrome (SARS) coronavirus which has also been causing outbreaks with high morbidity and mortality for the past 2 decades but with lesser global spread compared to COVID-19 (Drosten et al., 2003; Zaki et al., 2012). Notably, the number of infections and death associated with COVID-19 were reported to be 766,440,796 and 6,932,591 cases respectively as of 17 May 2023 (WHO, 2023). Sequencing of the SARS-CoV-2 genome has given us a better understanding of the virus’s organization (Figure 1), the virus has 29,890 base pairs of genes (GenBank NC 045512.2), which create 29 proteins and were encoded in 10 open reading frames.

The SARS-CoV-2 genome encodes four structural and sixteen non-structural proteins (NSPs), with both proteins performing varying biological activities in the virus. The structural proteins include the spike (S), membrane (M) protein, nucleocapsid (N), and envelope (E) proteins (Wang et al., 2017) while the NSPs include nsp7, nsp8, nsp12, and duplex of RNA-template product, Main protease Mpro or 3CL (3 chymotrypsin-like proteases) (Mori et al., 2020; Wu et al., 2022). The S protein is composed of two subunits S1 and S2 (Hillen et al., 2020; Mori et al., 2020; Petrosillo et al., 2020; Capasso et al., 2021), with the S1 subunit which contains the receptor-binding domain involved in the binding to the Angiotensin-converting 2 (ACE2) receptor on the host cell while the S2 subunit is responsible for membrane fusion (Satarker and Nampoothiri, 2020). The initiation of viral infection in the human body is the sequel to the entry of the virus via the ACE2 receptor, a phenomenon that reduces the ACE2 effect and leads to serious lung infection (Lu et al., 2020). Following the entry of the virus into the host cell, the viral RNA is translated into a polypeptide which is proteolytically cleaved into the sixteen NSPs of the virus by 3-chymotrypsin-like protease or main protease (Mpro) in concert with papain-like protease. The Mpro is pivotal to the sustenance of the life of the virus due to its mediation of the production of NSP4 to NSP16 (Umar et al., 2022). Interestingly, no homolog of this protein has been identified in humans. Hence, the S protein, the ACE2 receptor, and the Mpro have been attractive targets for COVID-19 drug development odysseys. So far, the widely utilized therapeutic strategy against COVID-19 is the administration of prophylactic and therapeutic vaccines, and this method has been effective in combating the infection. However, certain side effects including reactogenicity have been reported following their administration, while there has also been reluctance toward vaccination due to beliefs (Jafari et al., 2022). Hence, the search for potent antiviral drugs remains unfaltering, with many of the efforts aimed at identifying potential anti-SARS-CoV-2 drugs by exploring compounds from medicinal plants due to their chemical diversity and well-reported antiviral properties (Ali et al., 2021).

**FIGURE 2:**
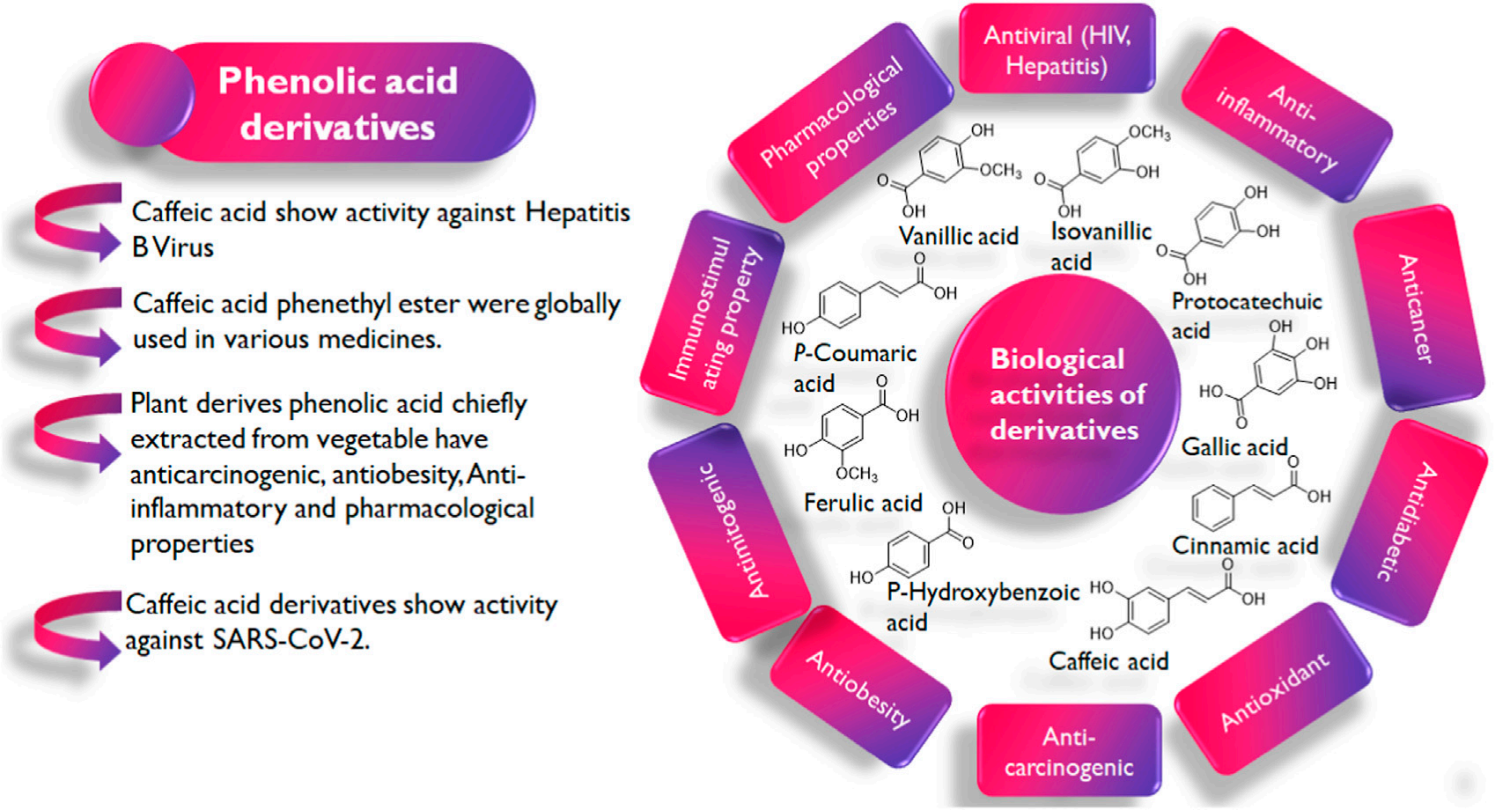
Pharmacological inspiration of phenolic acid derivatives.

In the exploration of medicinal plants with noteworthy pharmacological activities (Figure 2), a group of plant secondary metabolites, namely, the polyphenols have been recognized for their diversity, ubiquitousness, and potent biological activities (Laganà et al., 2019). The antiviral properties of this class of phytochemicals have also been reported in studies. Exemplifying this is the study by MV Kozlov et al. (2019) in which they synthesized cinnamic hydroxamic acids (CHA) and their ortho, para, and meta-substituted derivatives which were reported to show antiviral activity against the Hepatitis C virus. Specifically, the meta-substituted CHAs inhibit the replication of the genetic material of the virus but the para-substituted CHAs were reported to show stronger inhibitory activities.

Interestingly, phenolic acids and their derivatives have also been explored in the ongoing fight against COVID-19 with several studies reporting their efficacy in both *in vitro* and *in vivo* studies (Tirado-Kulieva et al., 2022). Rosmarinic acid’s IC50 value for inhibiting Mpro is 6.84 µM. As opposed to the initial theory that orthoquinone synthesis came from oxidizing the phenolic hydroxyl groups of rosmarinic acid, the mechanism suggested by Krüger et al. (2023) proposes a covalent modification of Mpro contacts between Cys145 and the Michael acceptor donor. However, the exploration in some studies is often limited to the search for monotargeted drugs and not multitargeted drugs capable of exerting their anti-SARS-CoV-2 activity via more than one of its proteins.

**FIGURE 3:**
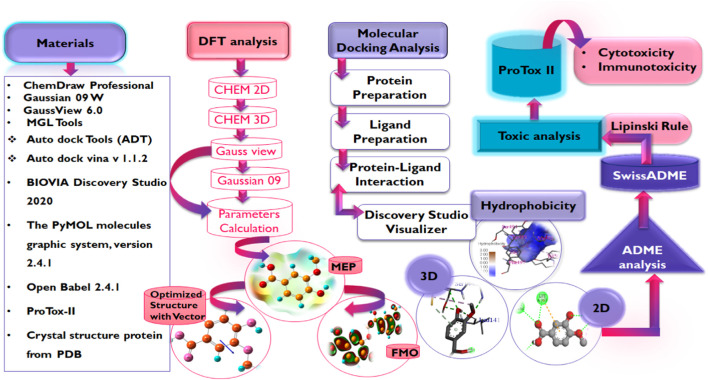
Workflow for study.

In light of this evidence, this study aims to evaluate the potential of phenolic acids and their derivatives to function as inhibitors of the critical proteins in the life cycle of SARS-CoV-2 using molecular modeling techniques including molecular docking, DFT, and MD simulation studies. It is worth noting that advances in bioinformatics have enabled the rapid identification of potential drug candidates, hence, reducing the time and cost associated with drug discovery and development. Hence, the reason for its utilization in this study Figure 3.

## 2 Methodology

### 2.1 Retrieval of compounds

A library of naturally occurring phenolics (Supplementary Table S1) was assembled by searching literature-based databases such as Google Scholar, Scopus, PubMed PubChem, and online libraries like Zinc, Coconut, with keywords like natural products, phenolic acids, FDA-approved and traditional herbal medicines (Singla et al., 2021). Recent literature was surveyed from 2019 to 2022 for retrieval of compounds by exempting the articles non-peer-reviewed, short reports, and communication letters having no PubMed Identification and Digital Object Identifier (DOI) for assurance of the credibility of the literature study (Pamuru et al., 2020).

### 2.2 Software used for structure-based virtual screening of compounds

The crystal structures of the proteins were retrieved from the protein databank (Hatada et al., 2020) while the structures of the compounds were obtained by drawing and optimization using ChemDraw Professional (Version 19.1.0.8, Perkin Elmer, Waltham, WIA, United States), and Open Babel 2.4.1 (Yoshikawa and Hutchison, 2019) used for formatting the ligand file (F Ghazi et al., 2021). MGL Tools (1.5.7) (Mohapatra et al., 2021), Auto dock Tools (ADT), Python Molecular views (PMV), Auto dock vina v 1.1.2 (Trott and Olson, 2010), BIOVIA Discovery Studio 2020 (v20.1.0.19295) were used for molecular docking studies along with visualization (Braz et al., 2020), PyMOL molecules graphic system, version 2.4.1 (Schrodinger, LLC) provide space for validation of docking via re-docking approach (Yuan et al., 2016), and ProTox-II server for toxicity prediction (Banerjee et al., 2018). The electronic properties of the compounds were studied via the DFT using Gaussian 09 W (Mohapatra et al., 2021), and GaussView 6.0 (Tomberg, 2013).

### 2.3 Molecular docking

#### 2.3.1 Protein preparation

**FIGURE 4:**
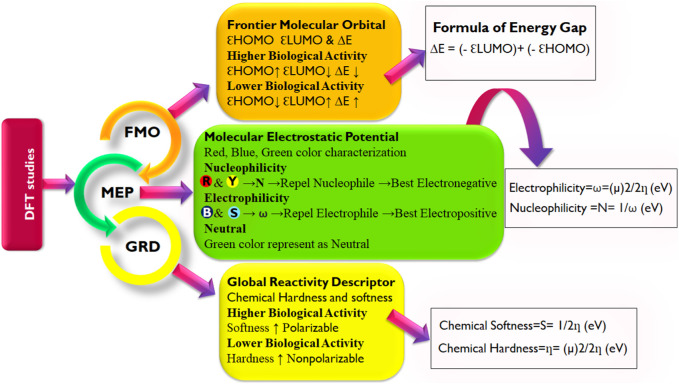
Methodology employed for DFT analysis.

Three-dimensional structures of the proteins in crystal format were retrieved from the RCSB protein Data Bank (https://www.pdb.org) in PDB format (PDB ID: 6LU7, 6LZG) (Jin et al., 2020; Nadjiba et al., 2020) and were imported into the interface of Discovery Studio Visualizer v20. 1. 0. 19295 (Accelrys). During the preparation of protein, the SBD sphere was generated on the active site of protein, this part of protein has no missing residue and it is safe for target ligand to bind at this site (also known as active site) of the protein. All the water molecules present in the protein were deleted. The binding sites were determined by structure based design (SBD) utilizing the space occupied by the co-crystallized ligand (Delmondes and Delmondes, 2018). Subsequently, the co-crystallized ligands were deleted prior to molecular docking purposes and polar hydrogens were added to the protein for protonation. Conformers were generated by uploading ligand in pdbqt format to Auto Dock Vina for protein-ligand interaction accompanied by scoring function for generated conformers, out of which best docked conformer or pose is selected for further analysis. Thee prepared structures of the proteins were saved in PDB format which was then converted into a PDBQT file by Autodock Tools v.1.5.7 (Sharma and Sarkar, 2012; Shen et al., 2013; Delmondes and Stefani, 2018). Table 1 presents some information on the selected proteins Figure 4.

**TABLE 1:** Basic information of the receptor molecules used for molecular docking.

PDB ID	Proteins	Co-crystallized ligands	Resolution (Å)
6LU7	3C-like proteinase	N-[(5-methylisoxazol-3-YL) carbonyl] alanyl-L-vanyl N∼1∼-[(1R,2Z)-4-(benzyloxy)]-4 OXO-1-{[(3R)-2 oxopyrrolidine -3-YL] methyl}-BUT-2-ENYL-L-leucinamide	2.16
6LZG	Spike protein S1	2-acetamido-2-deoxy-beta-D-glucopyranose	3.46

#### 2.3.2 Ligand preparation

The ligands were drawn by using ChemDraw Professional 19.1.0.8, then the three-dimensional structure was obtained by Chem3D 19.1, energy minimization and optimization was achieved by applying MM2 and MMFF94 force fields, and finally, the structure was saved in PDB format and converted into PDBQT format by using Open Babel, as it was a requirement of AutoDock Vina docking. Then by using AutoDock Tools v. 1.5.7 all the Kollman and Gastieger charges were added by assigning AD4-type atomic radii (Shen et al., 2013; Sadati et al., 2019). Ultimately, the prepared structures of the ligands were docked into the active sites of proteins using the AutoDock Vina software program (Azam and Abbasi, 2013).

#### 2.3.3 Protein-ligand interaction

Start Windows by typing “cmd” into the Start menu to launch the Command Prompt. To access the directory holding your docked files, use the “cd” command. The AutoDock Vina configuration file should be run. To ensure precise docking, modify the configuration file (config.txt) to include the appropriate SBD. Set the “size_x,” “size_y,” and “size_z” parameters to describe the dimensions and the “center_x,” “center_y,” and “center_z” parameters to define the sphere’s center.

AutoDock Vina creates output files after the docking is finished. Using the proper file naming conventions, save the results in PDBQT format. Parse the docking output file using a scripting language like Python to separate the conformers or postures according to their scores. Identifying candidates with higher binding affinities will be made easier by doing this. Based on the docking scores, order the separated postures. The position with the highest binding affinity has the lowest score. Recognize and take note of the ligand-receptor interactions in this position.

Following the molecular docking of the ligands into the active site of the proteins, the complexes formed were imported to Discovery Studio Visualizer v20. 1. 0. 19295 (Accelrys) to delineate the interaction between the ligands and the proteins in 2D and 3D poses.

#### 2.3.4 Drug likeness test and pharmacokinetics analysis

The druglikeness of the hit compounds was evaluated based on Lipinski’s rule of five (Ro5) via the AdmetSAR 2.0 webserver (http://lmmd.ecust.edu.cn/admetsar2). Subsequently, the pharmacokinetics parameters of the compounds were studied via the same webserver while the ProTox-II webserver (https://tox-new.charite.de/protox_II) (Adem Ş. et al., 2021) was employed to extensively study the toxicity profiles of the compounds.

### 2.4 Optimization of compounds by density functional theory (DFT) model

Density functional language is a universal approach for obtaining the electronic properties of organic compounds and their activity relationships by making use of quantum chemical descriptors and other reactivity parameters (Kohn et al., 1996). The main objective is the correlation of the biological activities of compounds with molecular descriptors given by DFT calculations (Farag and Fahim, 2019). All computational calculations of compounds were performed using the Gaussian 09W program supported by the Gauss View 6.0.16 which was used to optimize the structures (Mishra and Srivastava, 2014). The hit compounds obtained from this study that were also found to possess good druglikeness and pharmacokinetics properties were subjected to the calculation. All compounds were first drawn in ChemDraw Professional (Version 19.1.0.8, Perkin Elmer, Waltham, WIA, United States) in 2D form, then the structures were copied and pasted into CHEM 3D, a software of ChemDraw Professional, for MM2 and MMFF94 minimization. After minimization in CHEM 3D, the structures of the compound were saved into mol2 file, this mol2 file was then opened in the GaussView 6.0 interface and subjected to further optimization and to calculate density functional parameters as FMO, GRD and MEP. The DFT calculations were performed using the B3LYP functional using the 6-311G basis set to describe the electronic structure of the compounds (Ozdemir et al., 2015).

### 2.5 Molecular dynamic simulation analysis

To study the stability of the complexes formed following the docking simulation, the hit compounds with desirable druglikeness and pharmacokinetics properties were subjected to molecular dynamics simulation using the online server IMODS (Sumera et al., 2022). To achieve this, the PDB file of the resulting complexes obtained from docking was uploaded to the server (Kirar et al., 2022).

Open protein on the Biovia Discovery Studio, ligand of protein with the heaviest atoms saved in a separate window. After that the sequencing and alignment of protein done, the saved ligand again inserted in the prepared protein. From the display tab go in the structure and select the RMSD and further select the heavy atoms and RMDS will be displayed.

## 3 Results and discussion

### 3.1 Molecular docking study of SARS-CoV-2 structural protein

**FIGURE 5:**
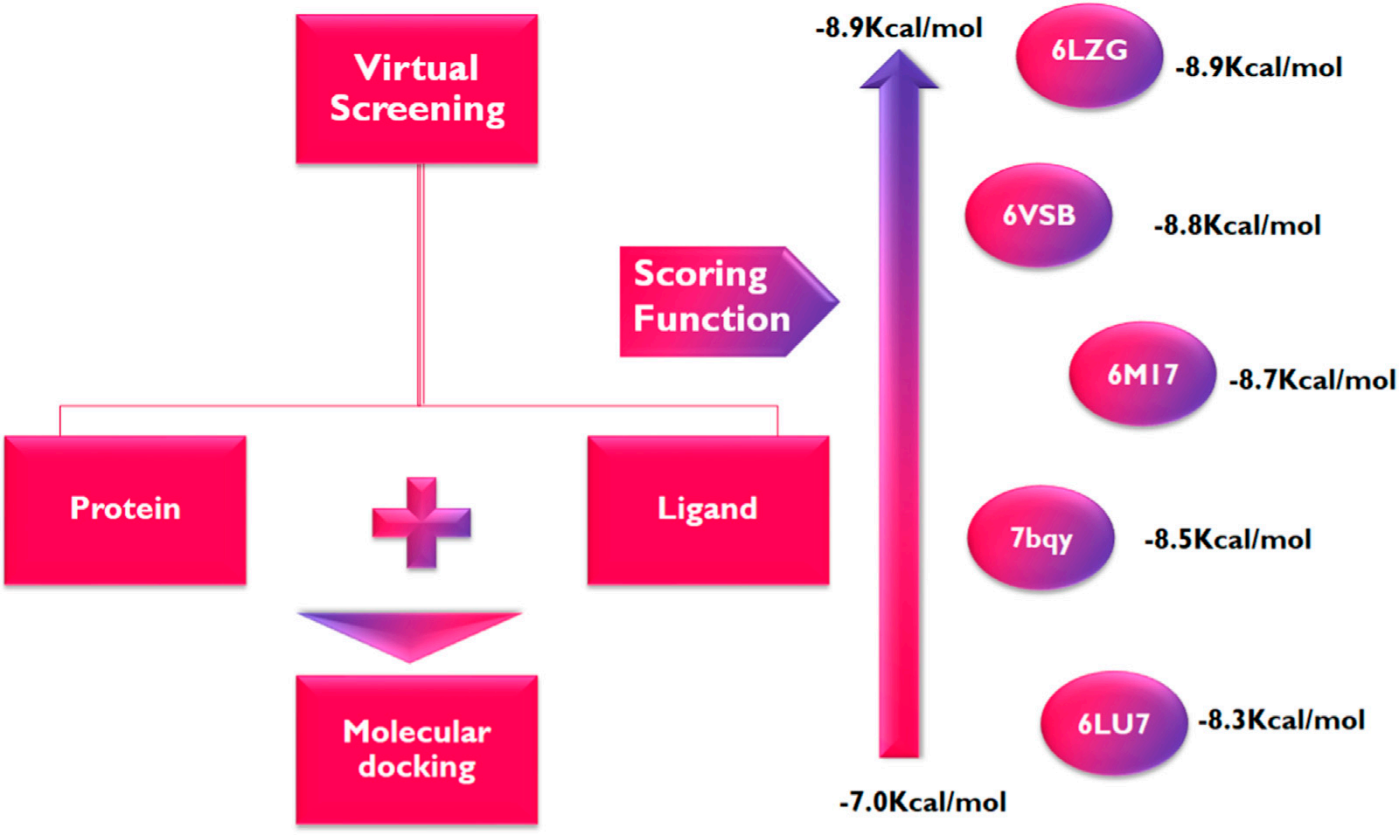
Parameters utilized for docking analysis.

Following the identification and retrieval of the structures of the thirty-five phenolic acid compounds (Supplementary Table S1), the structures of the proteins were also retrieved from PDB, and both were prepared as described above. Subsequently, the compounds were docked into the active sites of the proteins to identify the interactions and decipher the binding affinities of each compound for the protein. Noteworthy, Remdesivir, which was used as the Standard drug for the docking study was also docked against the proteins. To identify the hit compounds following molecular docking (Figure 5), a filtering criterion was applied and compounds with docking scores ranging from −7.0 to −8.9 kcal/mol were considered the hit compounds. Noteworthy, a lower docking score indicates a better binding affinity. The docking scores of the hit compounds against their respective proteins are presented in Table 2 while the docking scores of the remaining compounds are presented in Supplementary Table S2. As evident in Table 2, the hit compounds against the Mpro had docking scores ranging from −7.3 to −8.5 kcal/mol while the standards had a docking score of −7.8 kcal/mol. Table 2: docking score of hit compounds as filtering criteria.

**TABLE 2:** docking score of compounds as filtering criteria.

Compound name PubChem ID, drugbank accession number	PDB ID: 6LU7	Compound name PubChem ID, drugbank accession number	PDB ID: 6LZG
	Docking score		Docking score
Coutaric acid (A26) 57517924	−7.5	Fertaric acid (**A25**) 22298372	−7.6
Sibirioside A (A30) 6326022	−7.3	Chlorogenic acid (**A31**) 1794427	−8.2
Chlorogenic acid (A31) 1794427	−7.3	*P-*Coumaric acid glucoside (**A32**) 8016010	−8.0
Rosmarinic acid (A33) 5281792	−7.4	Rosmarinic acid (**A33**) 5281792	−8.2
Chicoric acid (A34) 5281764	−7.4	Chicoric acid (**A34**) 5281764	−8.2
Cynarine (A35) 5281769	−8.5	Cynarine (**A35**) 5281769	−8.9
Remdesivir DB14761	−7.8	Remdesivir DB14761	−8.2

Interestingly, only Cynarine with a docking score of −8.5 kcal/mol was found to possess a higher docking score than that of the Remdesivir while other compounds including Coutaric acid, Sibirioside A, Chlorogenic acid were also among the hit compounds against the protein. Similarly, set of hit compounds against the S protein subunit 1 was found to have docking scores ranging from −7.6 to −8.9 kcal/mol, while Remdesivir had a docking score of −8.2 kcal/mol. Interestingly, cynarine was also found to be the compound with the highest binding affinity for this protein while Chlorogenic acid, Rosmarinic acid, and chicoric acid were also among the hit compounds. Validation of the docking was done to confirm the molecular docking results and protocol utilized in this study was performed by redocking of the co-crystalized ligands of each protein against their active sites and subsequently comparing their binding poses to that of the undocked native ligand to determine the root mean square deviation (RMSD). Process of redocking done by the docking of proteins with their native ligands (ligands of protein). The protein 6LU7 has only one native ligand that consist of 49 atoms. If a protein has more than one ligand then the ligand with the greatest number of atoms will be on priority. Protein 6LZG has four ligands (three ligand A and one ligand B) each one consist of fourteen atoms. First the process of redocking done with the series of 3 A ligands but the results were not satisfied that’s why the selected ligand for the purpose of redocking is ligand B (native ligand) in order to get the satisfied results as in Table 3 of redocking.

**TABLE 3:** Docking validation of protein 6LU7 and 6LZGalong with RMSD values.

Protein PDB ID	Protein type	RMSD value
6LU7	Main protease	0.204
6LZG	ACE2	1.420

**FIGURE 6:**
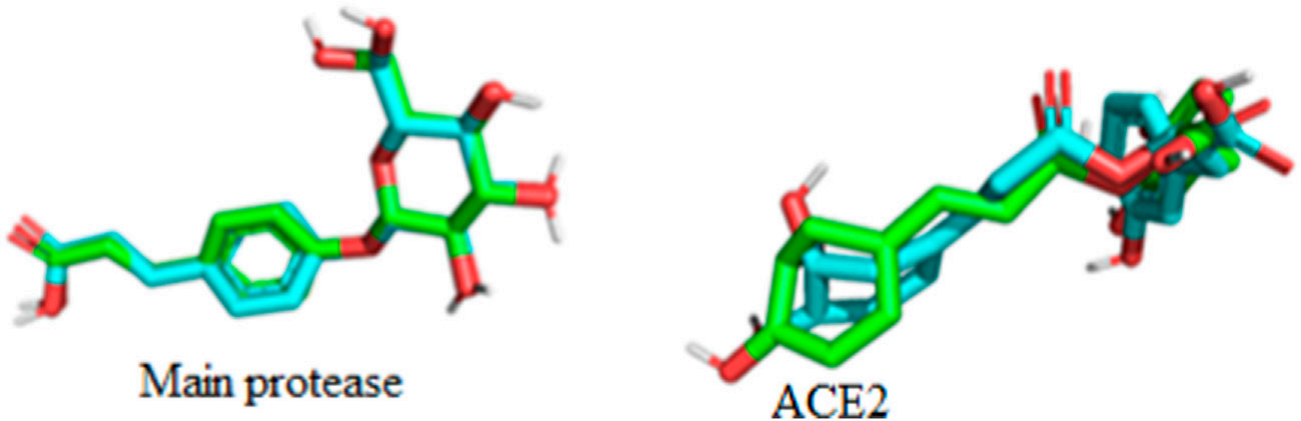
Superimposition and validation of docking.

The superimposition of the docked and undocked native ligand for each protein is depicted in Figure 6 while their RMSD values are presented in Table 3.

According to the result of (Table 3) protein 6LU7 is on lead as it has lowest RMSD value than the protein 6LZG. After the docking validation it has been proved that the protein 6LU7 is best for further screening.

### 3.2 Drug likeness and pharmacokinetics analysis

The evaluation of the drug and pharmacokinetics profile of a compound prior to preclinical and clinical trials have been one of the approaches to avoiding the failure of a potential lead compound, hence, leading to the identification of compounds that are worthy of further exploration and reducing the cost-burden of the drug discovery process. Consequently, the druglikeness and absorption, distribution, metabolism, excretion, and toxicity of the hit compounds derived in this study were profiled. The druglikeness of the compounds was evaluated based on the Ro5 which considers a compound as a potential oral drug if it fits in the following constraints: LogP < 5, MW < 500, no of rotatable bond (nRB) < 10, Hydrogen bond donor (HBD) < 5, and Hydrogen bond acceptor (HBA) < 10. A compound that violates more than one of the constraints of this parameter is considered non-druglike. Interestingly, of the four hit compounds for both proteins, only one compound, namely, cynarine was found to violate the Ro5 as evident in the result presented in Table 4. Specifically, the MW of cynarine with 516.45 da exceeded the set 500 da threshold, the HBA was found to be 11, and the number of HBD was found to be 7. Hence, rendering it non-druglike.

**TABLE 4:** Drug likeness properties of the hit compounds of Mpro and Ace2.

Compound	MW (da)	ALogp	HBA	HBD	Ro5 violation
Chlorogenic acid	354.31	−0.65	8	6	1
Rosmarinic acid	360.32	1.76	7	5	0
Chicoric acid	474.37	1.23	10	6	1
Cynarine	516.46	1.03	11	7	2
Remdesivir	418.49	4.30	5	0	0

Profiling of the pharmacokinetics of the hit compounds also revealed all the compounds as being absorbable by the human Intestine, they were all predicted to be non-Caco-2 permeant and not orally bioavailable (Table 5). Since the compounds were predicted to be absorbable by the human intestine, their inability to permeate the Caco-2 may be considered insignificant, also, the compounds that were predicted to be Ro5 compliant can be assumed to be orally bioavailable as opposed to the results of the prediction. The P-glycoprotein functions in the efflux or export of drug molecules out of cells, hence, probing the potentials of the drugs to inhibit or serves as the substrate of the protein is of great importance. All the compounds were found to be non-substrate of the protein while only chicoric acid was found to be an inhibitor of the protein (Table 5). These imply the compounds will not be exported out of the cell abruptly and there will not be any potential drug-drug interactions except during the administration of chicoric acid with other drugs that depend on the P-glycoprotein for their efflux. Further probing of the metabolism profiles of the compounds based on phase I metabolism mediated by CYP450 isozymes revealed the compounds possessed good profiles as evident by their inability to inhibit the studied isozymes. Of the four compounds, chlorogenic acid and cynarine were found to be substrates of CYP3A4 and CYP2C9. Conversely, Remdesivir was predicted to be a Caco-2 permeant and to be an inhibitor of the P-glycoprotein, it was also predicted to be an inhibitor of CYP2C19. Other properties were found to be similar to that of the hit compounds (Table 5).

**TABLE 5:** The ADMET profiles of the hit compounds of Mpro and ACE2 receptor.

Models	Chlorogenic acid	Rosmarinic acid	Chicoric acid	Cynarine	Remdesivir
Absorption and distribution					
Human intestinal absorption	+	+	+	+	+
Caco-2 permeability	—	—	—	—	+
p-glycoprotein (substrate)	—	—	—	—	—
P-glycoprotein (inhibitor)	—	—	+	—	+
Metabolism					
CYP2C9 (substrate)	+	—	—	+	—
CYP2C9 (inhibitor)	—	—	—	—	—
CYP2D6 (substrate)	—	—	—	—	—
CYP2D6 (inhibitor)	—	—	—	—	—
CYP3A4 (substrate)	+	—	—	+	—
CYP3A4 (inhibitor)	—	—	—	—	—
CYP1A2 (inhibitor)	—	—	—	—	—
CYP2C19 (inhibitor)	—	—	—	—	+
Toxicity					
Mutagenicity	—	—	—	—	—
Hepatotoxicity	—	—	—	—	—
Immunotoxicity	+	+	+	+	—
Toxicity class	5	5	5	5	6
Cytotoxicity	—	—	—	—	—
hERG inhibition	—	—	—	—	+

An extensive study of the toxicity profiles of the hit compounds was conducted using the ProTox-II server and the results revealed the compounds belonged to toxicity class 5 while the standard compound belonged to class 6, hence, suggesting that only an extremely high dosage of the compounds will be able to initiate a toxic response in humans (Table 5). Interestingly, all the compounds were also found to non-carcinogenic, non-hepatotoxic, non-mutagenic, and non-cytotoxic, however, they were all predicted to be immunotoxic except the Standard compound, hence, suggesting the need for further studies to probe into their specific effect on the immune system.

### 3.3 Molecular interaction profiling

**FIGURE 7:**
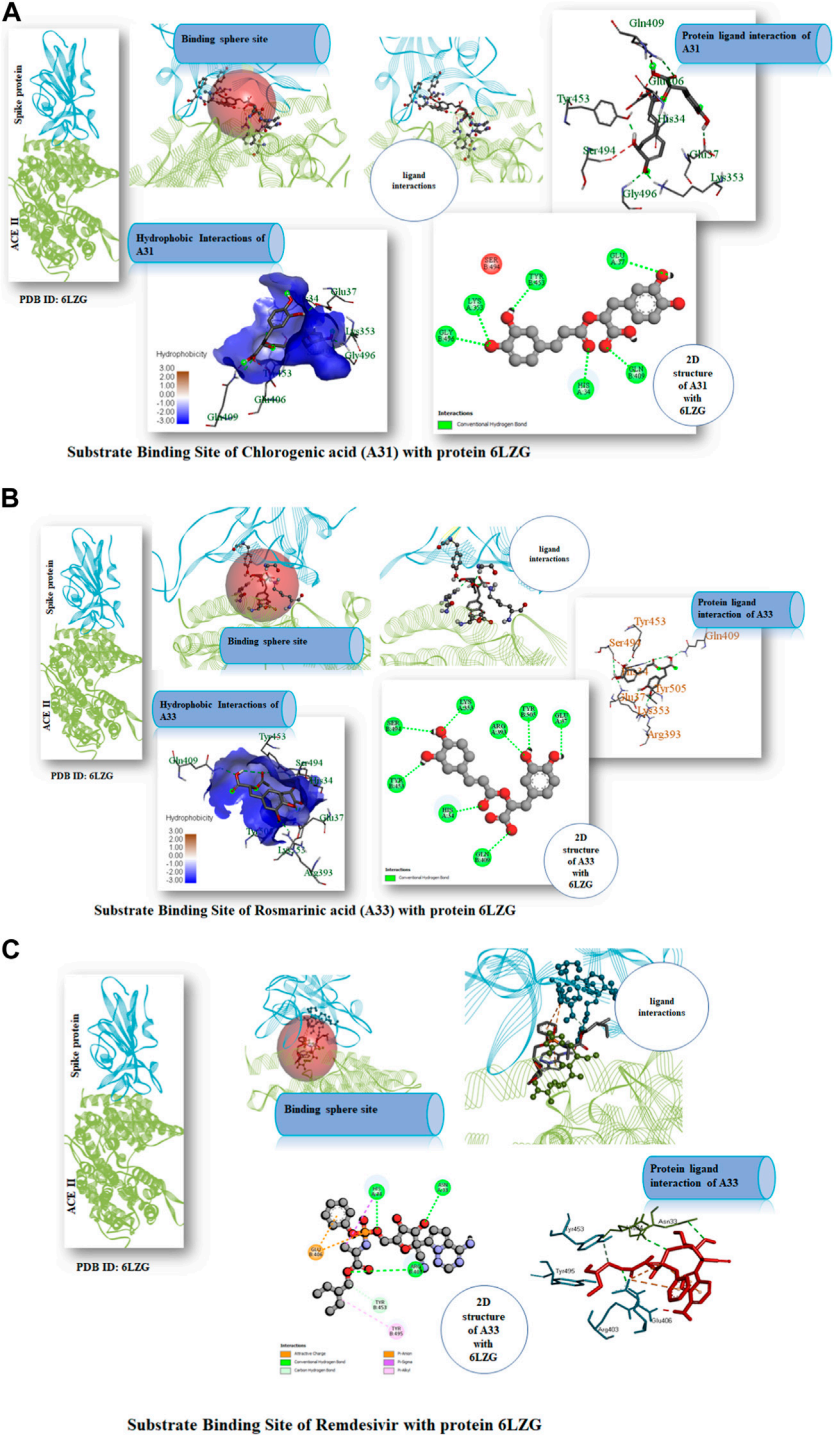
**(A)** Substrate Binding Site of Compound A31 (Chlorogenic acid) with protein 6LZG. **(B)** Substrate Binding Site of Compound A33 (Rosmarinic acid) with protein 6LZG. **(C)** Substrate Binding Site of Remdesivir with protein 6LZG.

Following the ascertaining of the druglikeness and suitability of the pharmacokinetics profiles of the hit compounds, the interaction of the compounds that were druglike and with good pharmacokinetics profile with the SARS-CoV-2 target protein were studied from the complex formed after molecular docking. Chlorogenic acid (**A31**) majorly interacted with the RBD via hydrogen bonds. Interestingly, hydrogen bond formation has been reported to be important to the selectivity and the optimization of compounds structures to maximize interactions with target proteins is often practiced in rational drug design (Gancia et al., 2001; Ghiandoni and Caldeweyher, 2023). Hence, suggesting that chlorogenic acid (**A31**) will strongly and stably bind to the protein. A Similar trend was noticed in the interaction of rosmarinic acid and chicoric acid with the protein. Analysis of docking results showed that chlorogenic acid and rosmarinic acid possessed high binding affinities for the residues in the active site of the SARS-CoV-2 proteins being studied. The result of docking analysis in Supplementary Table S2 showed that chlorogenic acid forms hydrogen bonding with HIS34 amino acids LYS353, GLN409, GLY496, TYR453, and GLU37 (Figure 7A). The rosmarinic acid formed 7 hydrogen bonds that interrelate with HIS34 amino acids LYS353, ARG393, GLN409, SER494, TYR453 and TYR505 (Figure 7B). The standard drug remdesivir docked with the protein 6LZG formed a conventional hydrogen bonding with ARG408 amino acid HIS34, ARG403 (Figure 7C). However, binding affinity result of the compound based on the number of hydrogen bond that formed with the ACE2 active site (Wang et al., 2022). The chlorogenic acid and rosmarinic acid showed high binding potential as compared to the reference Remdesivir. The good binding affinity of the compound depends on the number of bonding that occurs with the protein’s active site. Chlorogenic acid and rosmarinic acid showed many chemical interactions with 6LZG. These two compounds, chlorogenic acid and rosmarinic acid, were docked against the SAR-Cov 2 protein 6LZG and it was noted these compounds showed a good binding affinity and appreciable hydrogen bonding interactions with amino acid residues of the protein (Adem S. E. et al., 2021; Jahan et al., 2021; Patel et al., 2023).

### 3.4 Quantum reactivity analysis

The quantum reactivity of the hit compounds with desirable druglike and pharmacokinetics profiles was investigated via DFT calculations using the Gaussian software. The energies of the frontier molecular orbitals, namely, the highest occupied molecular orbital (HOMO) and the lowest unoccupied molecular orbitals (LUMO) were generated via the calculations and the results are presented in Table 6. Other quantum reactivity parameters including electron affinity, chemical hardness (η), chemical softness (ζ), electronegativity (χ), ionization potential, electronic chemical potential (µ), and electrophilicity index (ω) were calculated based on the values of the E_HOMO_ and E_LUMO_ according to Koopman’s theorem [30]. The equations utilized for the estimation of the reactivity parameters are:

$$\text{Energy Gap }\Delta E={HOMO}_{\varepsilon }-{LUMO}_{\varepsilon }$$ (1)

$$\text{Ionization Potential }I=-E_{HOMO}$$ (2)

$$\text{Electron affinity }A=-E_{LUMO}$$ (3)

$$\text{Chemical hardness }\eta =\frac{1}{2}\left(\frac{\partial^{2}E}{\partial N^{2}}\right)V=\frac{1}{2}\left(\frac{\partial µ}{\partial N}\right)V=\left(I-A\right)/2$$ (4)

$$\text{Softness }\zeta =\frac{1}{\eta }$$ (5)

$$\text{Electronegativity }\chi =-\mu =-\left(\frac{\partial E}{\partial N}\right)V=\left(I+A\right)/2$$ (6)

$$\text{Chemical potential }\mu =\left(\frac{\partial E}{\partial N}\right)V=-\left(I+A\right)/2$$ (7)

$$\text{Electrophilicity index }\omega =\frac{\mu 2}{2\eta }$$

**TABLE 6:** The computed values of the quantum mechanical properties of the druglike hit compounds and the standard compound.

S/N	Quantum chemical property	Compounds			
		Chlorogenic acid	Rosmarinic acid	Chicoric acid	Remdesivir
1	HOMO	0.229 eV	0.220	0.239	0.220
2	LUMO	0.080 eV	0.105	0.091	0.044
3	Energy gap (∆E)	0.149 eV	0.115	0.147	0.176
4	Ionization potential (I)	−0.229 eV	0.220	0.239	0.220
5	Electron affinity (A)	−0.080 eV	0.105	0.091	0.044
6	Chemical hardness (η)	0.074 eV	0.057	0.193	0.065
7	Chemical softness (ζ)	6.7132 eV	8.698	2.587	7.584
8	Electronegativity (𝜒)	0.284 eV	0.162	0.156	0.154
9	Chemical potential (𝜇)	−0.284 eV	−0.162	−0.156	0.154
10	Electrophilicity index (𝜔)	0.210 eV	0.229	0.169	0.181

**FIGURE 8:**
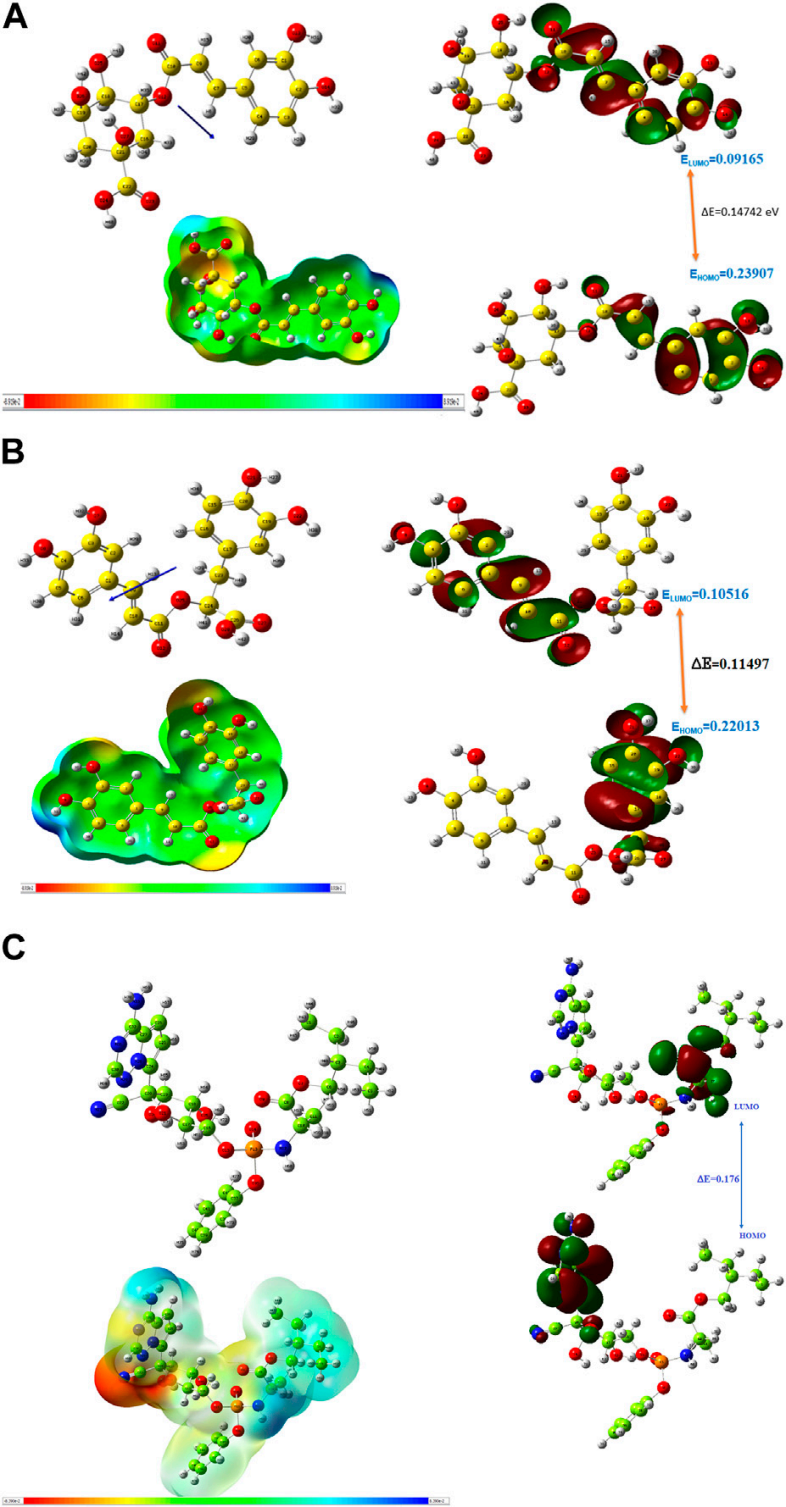
**(A)** Optimized structure, MEP and HOMO, LUMO parameters of chlorogenic acid. **(B)** Optimized structure, MEP and HOMO, LUMO parameters of Rosmarinic acid. **(C)** Optimized structure, MEP and HOMO, LUMO parameters of Remdesivir.

The HOMO energy value measures the amount of energy required to remove an electron from the HOMO orbital of a molecule, and it depicts the reactivity of the molecule in a chemical reaction. The LUMO energy value is the energy of the lowest energy orbital in a molecule which is deficient of an electron. The energy difference between the energy values of the HOMO and LUMO is referred to as the band gap energy and it gives insights into the stability and reactivity of a molecule and determines the amount of energy required to excite an electron from the HOMO to the LUMO. As evident in Table 6, rosmarinic acid was found to possess the lowest HOMO energy among the hit compounds, with chlorogenic acid and chicoric acid having the penultimate and the ultimate HOMO energy values respectively. Conversely, chlorogenic acid was found to possess the lowest LUMO energy value with chicoric acid and rosmarinic acid having the second highest and the highest energy values respectively. These results reveal that rosmarinic acid will be the most reactive and the least stable among the compounds and is expected to be more reactive than the standard drug (Remdesivir). Further supporting this inference is the band gap energy values of rosmarinic acid which was estimated to be the lowest of all the hit compounds (Figure 8). Hence, depicting the high potential of the compound to readily react with its targets could correspond to better pharmacological activity. Similarly, the band gap energy values also reveal that chicoric acid and chlorogenic acid will be the second-most table and the most stable compounds respectively. It is worth that chlorogenic acid and chicoric acid also exhibited electronic properties which are desirable as evident by the similarity of their values to that of the Remdesivir and that of chlorogenic acid. Also, other reactivity parameters including ionization potential (I), chemical potential (µ), electrophilicity index (ω), chemical hardness (η), and chemical softness (ζ) are in accordance with the inference as regards the stability and reactivity of the compounds as noted above.

**FIGURE 9:**
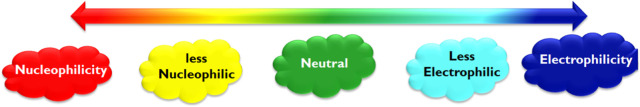
Molecular Electrostatic potential graph.

The most important global reactivity is the molecular electrostatic potential. The molecular electrostatic potential (Figure 9) plays a significant role in portending the reactive site for electrophilic and nucleophilic attack. The positive electrostatic potential is blue and the negative electrostatic potential is red. The MEP scale consists of three colors red color indicates the site for nucleophilic attack. The blue color indicates the site for electrophilic attack and the green color indicates the neutral region.

### 3.5 MD simulation

For the confirmation of the stability of the protein-ligand complexes, MD simulation was performed by using an online server iMOD. All the protein-ligand complexes including 6LU7-A33 were subjected to MD simulation to ascertain the stability of the protein-ligand complexes. According to the iMOD parameters, lower the eigenvalue higher will be the protein-ligand interaction as shown in Table 7.

**TABLE 7:** Eigen value for complexes.

Complexes	Eigenvalue
6LU7-A33	1.21 × 10^−4^

**FIGURE 10:**
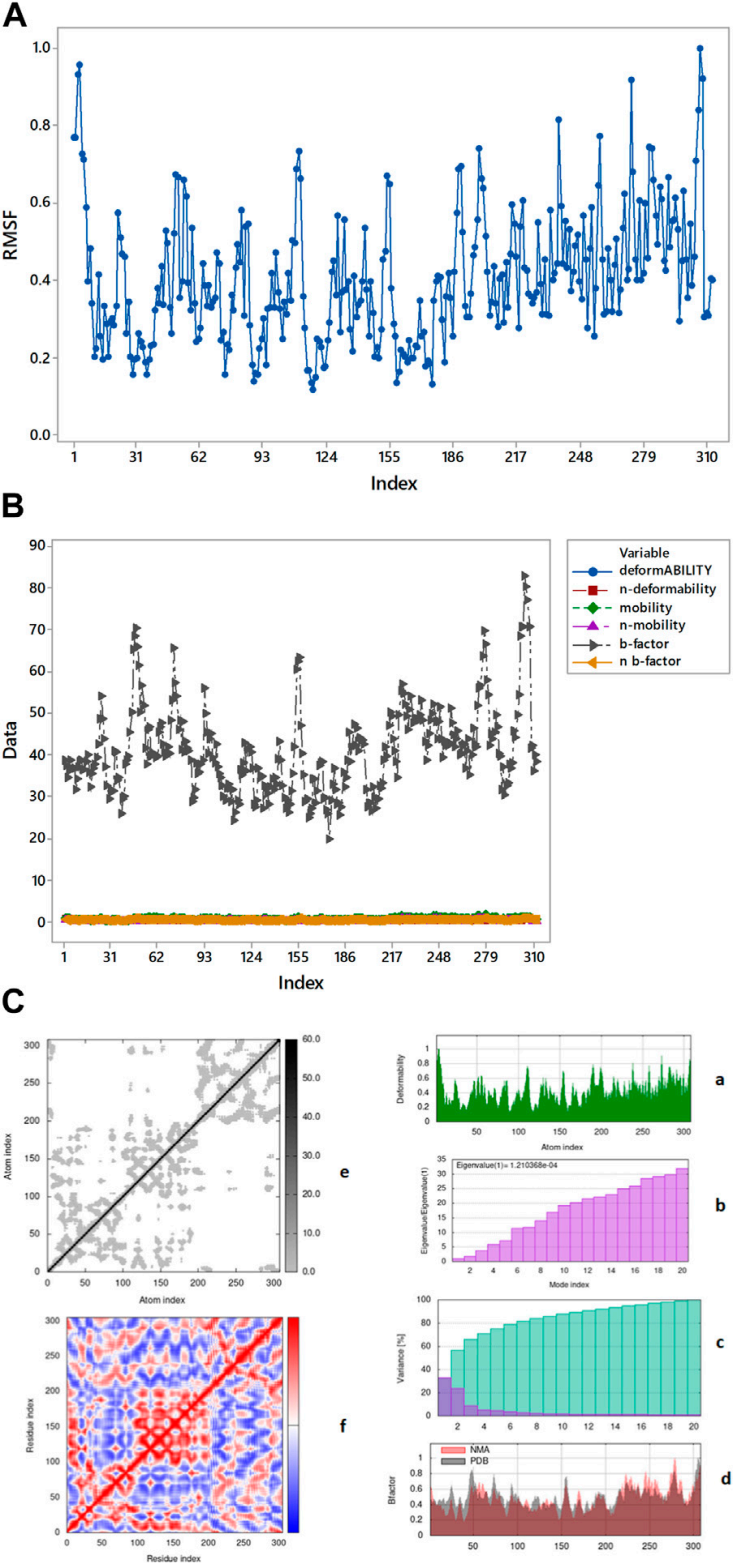
**(A)** Root means square fluctuation of 6IU7-A33 complex presenting its movement around average position. **(B)** RMSF value OVERLAPPING. **(C)** Graphical representation of complex 6LU7-A33 (a) deformability (b) eigenvalue (c) variance (d) B-factor elastic model network (e) overall stiffness (f) covariance map.

Complex 6lu7-A33 (Figures 10A, B) is the best complex due to the low eigenvalue and RMSF value for most of residues. Lower RMSF of residues indicated restricted movements around the average position during the simulation. For the investigation of slow dynamics and conformational fluctuations, normal mode analysis (NMA) plays an important role.

In the process of md simulation the distance of ligand from the active site of protein calculated by IMODS (online server) as well as the distance before and after simulation is shown in table above. Protein 6LU7 has three chains (A, C, A), in Table 8 it has been shown that the chain A (1) has no distance or specific interactions with the ligand while chain A (2) has 45.48452 distance from ligand before simulation and after simulation distance is 0.686731. In chain C (3) distance from ligand from simulation is 42.12228 while after the simulation is 0.419554.

**TABLE 8:** Distance between the ligand and active sites of protein.

Chains	Before	After
1	0	0
2	45.48452	0.686731
3	42.12228	0.419554

By iMOD server these protein-ligand complexes give mobility profiles such as B-factor as well as deformability (Sumera et al., 2022). The high B-factor (Figure 10B) shows greater flexibility and thermal motion, while a lower B-factor suggests less motion and a more ordered structure. The graphical representation (Figure 10C) of the complexes 6LU7-A33 for the deformability factors shows that higher the peaks higher will be the deformability of the complex. B-factor is obtained by a comparison of the value derived for that of protein data bank (PDB) structures and that of the normal mode analysis (NMA) performed on the complex.

The covariance factor of the complex 6LU7-A33 displays correlation between pair of residues of the complex. The red color shows a very correlation between protein and ligand whereas the white color displays no correlation. The blue color in the graph displays the anti-correlation of protein-ligand complex. It is worth noting that a good correlation corresponds to a good complex. Hence, the complex studied can be inferred to be a stable one. The darker shaded grey area in the graph represents the overall stiffness of the complex as shown in Figure 10C (e) (Santra and Maiti, 2022).

## 4 Conclusion

Herein, this study evaluated the potentials of thirty-five phenolic acid derivatives to serve as dual inhibitors of two proteins that are critical to the life cycle of SARS-CoV-2 using molecular modeling techniques including molecular docking, DFT calculations, and molecular dynamics simulation among many others. Following an extensive screening of the compounds via subjection to a pipeline that was aimed at identifying the lead compounds among the studied compounds, rosmarinic acid, chicoric acid, and chlorogenic acid were discovered to be the best set of compounds derived in this study. Notably, these compounds possessed high binding affinities for the proteins and also possessed good druglikeness and pharmacokinetics properties as well as other desirable properties including the formation of stable complexes with the protein and possessing pharmacological-suitable electronic properties which could contribute to their inhibitory potential, hence rendering worthy of exploration in further *in vitro* and *in vivo* studies aimed at developing anti-SARS-Cov-2 therapies.

## Data Availability

The original contributions presented in the study are included in the article/Supplementary Material, further inquiries can be directed to the corresponding authors.
