# Supplementary material for: Exploration of phenolic acid derivatives as inhibitors of SARS-CoV-2 main protease and receptor binding domain: potential candidates for anti-SARS-CoV-2 therapy
Source: Front Chem. 2023 Sep 26;11:1251529. doi: 10.3389/fchem.2023.1251529 (PMC10562575; doi:10.3389/fchem.2023.1251529)
Supplement: Supplementary file 1 [file Table1.docx]

**Exploration of Phenolic Acid Derivatives as Inhibitors of SARS-CoV-2 Main Protease and Receptor Binding Domain: Potential Candidates for Anti-SARS-CoV-2 Therapy**

**Nusrat Shafiq^1^*, Aiman Mehroze^1^, Warda Sarwar^1^, Uzma Arshad^1^, Shagufta Parveen^1^, Maryam Rashid^1^, Ariba Farooq^2^, Naila Rafiq^3^_,_ Fentahun Wondmie Gezahign*^4^, Yousef A. Bin Jardan^5^, Simone Brogi^6^ , Mohammed Bourhia^7^**

^1^Synthetic and Natural Products Discovery (SNPD) Laboratory, Department of Chemistry, Government College Women University Faisalabad-38000, Pakistan

^2^Department of Chemistry, University of Lahore, Lahore, Pakistan

^3^Department of Biochemistry, Government College Women University Faisalabad-38000, Pakistan

^4^ Department of Biology, Bahir Dar University, P.O.Box 79, Bahir Dar, Ethiopia

^5^ Department of Pharmaceutics, College of Pharmacy, King Saud University, P.O. Box 11451, Riyadh, Saudi Arabia

^6^Department of pharmacy, Pisa University, Italy

^7^Department of Chemistry and Biochemistry, Faculty of Medicine and Pharmacy, Ibn Zohr University, Laayoune 70000, Morocco

Correspondance : dr.nusratshafiq@gcwuf.edu.pk (NS) ; resercherfent@gmail.com ( FWG)

**Supplementary file**

**Table S1**: Phenolic acid derivatives with their structure, plant source, IC_50_ and molecular weight.

| **Sr#** | **Compound Name** | **Structure of compound** | **IC_50_ Value** | **Plant Source** | **Molecular weight (g/mol)** | **References** |
| --- | --- | --- | --- | --- | --- | --- |
| 1 | Vanillic acid (**A1**) |  | 7.45 mmol/L | *Angelica sinensis* | 168.14 | (Kim *et al.*, 2010, Bi *et al.*, 2010). |
| 2 | Isovanillic acid (**A2**) |  | 385.37 μg/mL | *Alphonsea cylindrica* | 168.15 | (Cho *et al.*, 2018). |
| 3 | Protocatechuic acid (**A3**) |  | 22.29 μg/mL | *Hibiscus sabdariffa* | 154.12 | (Alegbe *et al.*, 2019). |
| 4 | Syringic acid (**A4**) |  | <10^–12^ M | Quercus infectoria | 198.17 | (Srinivasulu *et al.*, 2018). |
| 5 | Gallic acid (**A5**) |  | 109.78 µg/mL | *Phyllanthus emblica, Terminalia bellirica* | 170.12 | (Genwali *et al.*, 2013). |
| 6 | Salicylic acid (**A6**) |  | 7.8 ± 0.3 µM | *Brassica rapa* | 138.12 | (Dhagat *et al.*, 2007, Thiruvengadam *et al.*, 2016). |
| 7 | Orselinic acid (**A7**) |  | 10.4 ± 0.5 µg/mL | *Rhododendron dauricum* | 168.15 | (Thadhani *et al.*, 2015, Taura *et al.*, 2016). |
| 8 | Olivetolic acid (**A8**) |  | < 100 μM | *Elephantopus scaber* | 223.24 | (ISMED *et al.*, 2019). |
| 9 | Ferulic acid (**A9**) |  | 9.9 µg/mL | *Cimicifuga heracleifolia* | 194.18 | (Ou and Kwok, 2004, Rivero-Cruz *et al.*, 2020). |
| 10 | Ethyl ferulate (**A10**) |  | 26.4 μM | Rubus corchorifolius | 222.24 | (Chen *et al.*, 2021). |
| 11 | 2,4,6 trihydroxybenzoic acid (**A11**) |  | 231 μg/mL | Anagallis arvensis | 170.12 | (Saleem *et al.*, 2020). |
| 12 | *p-*hydroxybenzoic acid (**A12**) |  | 7.6 ± 1.0 μg/mL | *Oberonia myosurus* | 138.12 | (Ren *et al.*, 2021). |
| 13 | Gentisic acid (**A13**) |  | 25.6 μM | Micronychia tsiramiramy | 154.12 | (Razakarivony *et al.*, 2016). |
| 14 | Shikimic acid (**A14**) |  | 6.5 ± 0.4 μg/mL | *Terminalia macroptera* | 174.15 | (Pham *et al.*, 2011). |
| 15 | Quinic acid (**A15**) |  | 53.82 μg/mL | Helichrysum mimetes | 192.17 | (Yazdi *et al.*, 2019). |
| 16 | Theogallin (**A16**) |  |  | Camellia sinensis | 344.27 | (Yang *et al.*, 2018). |
| 17 | Caffeic acid (**A17**) |  | 1.71–1.81 mg/mL | *Echinacea purpurea* | 180.16 | (Chiou *et al.*, 2017). |
| 18 | Cinnamic acid (**A18**) |  | 0.74±0.01 mM | Physalis angulata | 148.16 | (Moreira and de Souza Dias, 2018, Adisakwattana *et al.*, 2009). |
| 19 | Sinapinic acid (**A19**) |  | 8.97 pg/mL | ***Heliotropium strigosum*** | 224.21 | (Qayyum *et al.*, 2016). |
| 20 | Isoferulic acid (**A20**) |  | 7.30±0.57  μg/mL | *Rhizoma Cimicifugae* | 194.18 | (Wang *et al.*, 2011). |
| 21 | Umbellic acid (**A21**) |  | 124±5.76 µM | *Viola betonicifolia* | 180.16 | (Muhammad *et al.*, 2013). |
| 22 | Hydrocinnamic acid (phenylpropanoic acid) (**A22**) |  | 219.95 μg/mL | *Etlingera elatior* | 150.17 | (Grashella *et al.*, 2019). |
| 23 | *p-*coumaric acid (**A23**) |  | 2.3 ± 0.1 μg/mL | *Artocapus altilis* | 164.04 | (Nguyen *et al.*, 2012). |
| 24 | Phenethyl caffeate (**A24**) |  | 8 µM | *Rhodiola sacra* | 284.30 | (Imai *et al.*, 2019, Jung *et al.*, 2008). |
| 25 | Fertaric acid (**A25**) |  | 219 μg/mL | *Antidesma bunius* | 326.25 | (Funing *et al.*, 2021). |
| 26 | Coutaric acid (**A26**) |  | 8.48 μg/mL | \| \| \| \| \| *Lathyrus* \| \| --- \| \| \| --- \| --- \| \| \| --- \| --- \| --- \| \| \| --- \| --- \| --- \| --- \| \| \| --- \| --- \| --- \| --- \| --- \| | 296.23 | (Yazici *et al.*, 2020). |
| 27 | Caftaric acid (**A27**) |  | 10.21–169.3 µg/mL | *Echinacea purpurea* | 312.23 | (Hajimehdipoor *et al.*, 2012). |
| 28 | α-tocopherol (**A28**) |  | 0.19 mg/mL | *Cinnamomum cassia* | 430.71 | (Lin *et al.*, 2003). |
| 29 | Tocopheryl acetate (**A29**) |  | 0.109 µL/ mL | *Thymus vulgaris* | 472.7 | (Keramat *et al.*, 2017). |
| 30 | Sibirioside A (**A30**) |  | 13.5 μg/mL | *Scrophularia ningpoensis* | 472.2 | (Zhang *et al.*, 2017). |
| 31 | Chlorogenic acid (**A31**) |  | 0.11 mg/mL | *Cynara scolymus* | 354.31 | (Saleh *et al.*, 2016). |
| 32 | *p-*coumaric acid glucoside (**A32**) |  | 44 μg/mL | [*Heliophila coronopifolia*](https://www.sciencedirect.com/science/article/pii/S0031942211003669?casa_token=o0lg-0m_qNQAAAAA:u8XsfFvuZt0rA1UYkuOaYRQq4WqDAt380nce2C994EmSpZFH04VaCQRgk-GAXiZHWiGFGmWngw) | 326.29 | (Saito *et al.*, 2011). |
| 33 | Rosmarinic acid (**A33**) |  | 22.50±0.67 µg/mL | *Lamiaceae* | 360.31 | (Aicha *et al.*, 2020). |
| 34 | Chicoric acid (**A34**) |  | 0.28 mg/mL | *Echinacea purpurea* | 474.37 | (Kreft, 2005). |
| 35 | Cynarine (**A35**) |  | 1.85 mm/l | *Arnica montana* | 516.45 | (Danila *et al.*, 2016). |

**Table S2**: Depicted molecular interactions attained by following the rigid protein-ligand docking of target phenolic compounds with N3 inhibitor (PDB ID: 6LU7), rigid protein (PDB ID: 6LZG), rigid protein (PDB ID: 6VSB), rigid protein (PDB ID: 6M17) and rigid ligand-Protein (PDB ID: 7BQY) along with Standard drug Remdesivir

| PDB ID: 6LU7 | | | | | | | | | | | | | |  |  |
| --- | --- | --- | --- | --- | --- | --- | --- | --- | --- | --- | --- | --- | --- | --- | --- |
| Compound Name PubChem ID, Drugbank Accession number | Structure of Compound | | | Binding energy | | | Active site (Amino acid residue) | | | Protein-ligand interaction by H-bond | | | |  |  |
| Coutaric acid (**A26**) 57517924 |  | | | -7.5 | | | MET49 | | | TYR54, GLY143  CYS145, HIS163  GLU166, GLU166  LEU141, SER144  LEU141 | | | |  |  |
| Sibirioside A (**A30**) 6326022 |  | | | -7.3 | | | HIS41, MET49  MET165 | | | GLY143, SER144  CYS145, CYS145  GLU166, GLN189  MET49, GLN189  HIS41 | | | |  |  |
| Chlorogenic acid (**A31**) 1794427 |  | | | -7.3 | | | MET165, HIS41  CYS145 | | | HIS41, CYS145  HIS163, MET165  LEU141 | | | |  |  |
| Rosmarinic acid (**A33**) [5281792](https://pubchem.ncbi.nlm.nih.gov/compound/5281792) |  | | | -7.4 | | | Met165 | | | THR190, PRO168 | | | |  |  |
| Chicoric acid (**A34**) 5281764 |  | | | -7.4 | | | GLU166, CYS145 | | | GLY143, HIS163  GLN192, HIS164  ARG188, GLU166 | | | |  |  |
| Cynarine (**A35**) [5281769](https://pubchem.ncbi.nlm.nih.gov/compound/5281769) |  | | | -8.5 | | | MET49, CYS145 | | | TYR54, CYS145  HIS163, HIS164  LEU141, GLN189 | | | |  |  |
| Remdesivir DB14761 |  | | | -7.8 | | | HIS41, CYS145, CYS145, MET49, MET165, HIS41 | | | GLY143, SER144, CYS145, CYS145, CYS145, GLU166, LEU141, GLN189, GLU166 | | | |  |  |
| PDB ID: 6LZG | | | | | | | | | | | | | |  |  |
| ChemSpider, PubChem ID, Drugbank Accession number | | Compound Structure | | | | Binding energy | | | Active site (Amino acid residue) | | Protein-ligand interaction by H-bond | | | |  |
| Fertaric acid (**A25**) [22298372](https://pubchem.ncbi.nlm.nih.gov/compound/22298372) | |  | | | | -7.6 | | | ARG403, TYR495  PHE497, TYR505 | | HIS34, LYS353  PHE390, ARG393  ARG393, ARG393  GLY496, GLY496  GLN388, TYR495 | | | |  |
| Chlorogenic acid (**A31**) 1794427 | |  | | | | -8.2 | | |  | | HIS34, LYS353  GLN409, GLY496  TYR453, GLU37 | | | |  |
| *P-*Coumaric acid glucoside (**A32**) 8016010 | |  | | | | -8.0 | | | HIS34 | | HIS34, LYS353  ARG393, GLN409  SER494, SER494  TYR505, GLU37 | | | |  |
| Rosmarinic acid (**A33**) [5281792](https://pubchem.ncbi.nlm.nih.gov/compound/5281792) | |  | | | | -8.2 | | |  | | HIS34, LYS353  ARG393, GLN409  SER494, TYR453  TYR505 | | | |  |
| Chicoric acid (**A34**) 5281764 | |  | | | | -8.2 | | |  | | HIS34, LYS353  ARG393, GLY496  SER494, GLU37  GLU406 | | | |  |
| Cynarine (**A35**) [5281769](https://pubchem.ncbi.nlm.nih.gov/compound/5281769) | |  | | | | -8.9 | | | LYS353 | | ARG403, ARG408  ARG408, TYR505  ASP405, SER494  ARG403 | | | |  |
| Remdesivir DB14761 | |  | | | | -8.2 | | | GLU37, ARG408  HIS34, ARG403  TYR453, TYR495  TYR495, PHE497  TYR505, PRO389 | | ARG393, ARG393  ARG403, ARG403  ARG408, ARG408  GLN409, TYR505  GLU37, TYR505  HIS34, TYR453 | | | |  |
| PDB ID: 6VSB | | | | | | | | | | | | | | |  |
| **Compound Name, ChemSpider, PubChem ID, Drugbank Accession number** | | **Compound Structure** | | | | **Binding energy** | | | **Active site (Amino acid residue)** | | **Protein-ligand interaction by H-bond** | | | | |
| Sibirioside A (**A30**) 6326022 | |  | | | | -8.2 | | | ASP568, PHE855 | | ASN978, CYS743  ASP571, ASP571 | | | | |
| Chlorogenic acid (**A31**) 1794427 | |  | | | | -7.9 | | | ARG995, TYR756  ARG995 | | ARG995, ASP994  THR998 | | | | |
| *P-*Coumaric acid glucoside (**A32**) 8016010 | |  | | | | -8.2 | | | ARG995, TYR756  ARG995 | | ARG995, ARG995  ASP994 | | | | |
| Rosmarinic acid (**A33**) [5281792](https://pubchem.ncbi.nlm.nih.gov/compound/5281792) | |  | | | | -8.0 | | | ARG995, ARG995  ARG995 | | ARG995, ARG995  ASP994, ARG995  ASP994 | | | | |
| Chicoric acid (**A34**) 5281764 | |  | | | | -8.3 | | | TYR756, ARG995 | | TYR756, TYR756  ARG995, THR998  ASP994 | | | | |
| Cynarine (**A35**) [5281769](https://pubchem.ncbi.nlm.nih.gov/compound/5281769) | |  | | | | -8.8 | | | ARG355, TYR396  ASP198, GLY199  PRO230 | | SER514, PHE464  ASP198, GLY232  PHE515 | | | | |
| Remdesivir (DB14761) | |  | | | | -8.4 | | | GLU773, ARG1014, ARG1014, ARG765 ARG765, VAL772 GLU773, ILE1013, LEU1012, ARG765, ARG765, ALA766, LYS776 | | GLN954, ARG1019  ARG1019, GLN954 | | | | |
| PDB ID: 6M17 | | | | | | | | | | | | | | | |
| **Compound Name, PubChem ID, Drugbank Accession number** | | | **Compound Structure** | | **Binding energy** | | | **Active site (Amino acid residue)** | | | | **Protein-ligand interaction by H-bond** | | |  |
| Caftaric acid (**A27**) 6440397 | | |  | | -7.2 | | | LYS475 | | | | ARG482, ARG482  ARG482, TYR613  ASN674, GLU489  GLU495, GLU495 | | |  |
| α-tocopherol (**A28**) 14985 | | |  | | -7.7 | | | LYS475, VAL672, LEU675 | | | | GLU479 | | |  |
| Sibirioside A (**A30**) 6326022 | | |  | | -7.9 | | | LEU675 | | | | ARG644, ARG644  ARG644, VAL672  ASP494 | | |  |
| Rosmarinic acid (**A33**) [5281792](https://pubchem.ncbi.nlm.nih.gov/compound/5281792) | | |  | | -7.2 | | | GLU489, LYS475 | | | | HIS493, GLU495  ASN674, HIS493  TRP478 | | |  |
| Chicoric acid (**A34**) 5281764 | | |  | | -7.8 | | | ARG644, GLU667  LEU664 | | | | ARG652, ARG644  ARG644, ARG644  PRO492, ASP494  ASP637 | | |  |
| Cynarine (**A35**) [5281769](https://pubchem.ncbi.nlm.nih.gov/compound/5281769) | | |  | | -8.7 | | | ASP494, LEU664 | | | | LYS174, TYR497  ARG671, ARG671  VAL672, ASP637  ASP494, ARG644 | | |  |
| Remdesivir (DB14761) | | |  | | -8.3 | | | GLU479, GLU495  VAL672, LYS475  LYS475, LYS475 | | | | ARG482, ASN674  LEU675, LYS475  GLU489, VAL672  ASP494, ASN674 | | |  |
| PDB ID: 7BQY | | | | | | | | | | | | | | |  |
| Compound name, ChemSpider, PubChem ID, Drugbank Accession number | | | Compound structure | | Binding energy | | | Active site (Amino acid residue) | | | | | Protein-ligand interaction by H-bond | |  |
| Fertaric acid (**A25**) [22298372](https://pubchem.ncbi.nlm.nih.gov/compound/22298372) | | |  | | -7.0 | | | PRO168, MET165 | | | | | GLY143, THR190  PHE140, GLU166  ASN142 | |  |
| Caftaric acid (**A27**) 6440397 | | |  | | -7.1 | | | MET49 | | | | | TYR54, GLY143  SER144, CYS145  HIS163, SER144  PHE140, LEU141 | |  |
| Chlorogenic acid (**A31**) 1794427 | | |  | | -7.1 | | | MET49, HIS41  HIS41 | | | | |  | |  |
| Rosmarinic acid (**A33**) [5281792](https://pubchem.ncbi.nlm.nih.gov/compound/5281792) | | |  | | -7.2 | | | MET165 | | | | | GLU166, SER144  GLU166 | |  |
| Chicoric acid (**A34**) 5281764 | | |  | | -7.1 | | | MET165, MET49  HIS41 | | | | | THR190, THR190  GLU166 | |  |
| Cynarine (**A35**) [5281769](https://pubchem.ncbi.nlm.nih.gov/compound/5281769) | | |  | | -8.3 | | | HIS41, MET49  MET165 | | | | |  | |  |
| Remdesivir (DB14761) | | |  | | -7.6 | | | HIS41, MET165  HIS41, MET165  MET49 | | | | | HIS41, ARG188  THR190, GLU166  THR26 | |  |
